# Chapter I: Dysentery

**Published:** 1883

**Authors:** 


### DYSENTERY.

### Its History, Pathology, and Treatment.

Medical writers from the time of Hippocrates have treated of dysentery, and the variety of opinions they have entertained is but little less than the number of writers by whom the subject has been discussed. Three thousand years have not sufficed to settle the question for all whether it be really a disease of independent existence, or whether it be only an adjunct of some other more general form of evil. Those who have regarded it as an independent disease have had no agreement of views as to its true nature, while those who have contended for its derived status have failed equally to agree as to the origin to be assigned to it and the nosological relations in which it ought to be placed. From Hippocrates and Celsus, Aretæus and Galen, through the long list of those of the ancient and middle ages, as well as through that of the modern, down to the latest and best of the writers of our own day, nothing is established but a great diversity of opinion, though nearly all have tried their best to remove difficulties and differences. But even now, in works of the latest date and most complete character, the subject is discussed as an unsettled question, and *Bamberger* seems to come no nearer to its adjustment than Hippocrates or Galen. He says upon the nature and general nosological relations of this fatal disease there are not only the most various opinions expressed, but that these are in many instances no less than the exact opposites of each other. He ascribes this well-known fact in part to the different pathological systems and opinions advocated by different writers, and in part to the difference in times and circumstances in which the disease has been developed. The whole difficulty has grown out of an unwillingness on the part of these writers to concede to the disease an existence, and a nature outside and independent of their systems and opinions which by its visible phenomena it has never failed to assert. While this was clearly seen as to the systems and opinions of precedent writers, singularly enough, each was as blind to the fact in its application to his own as were all his predecessors. The profession seems to have been incapable of conceiving of disease outside of this circle of system, relationship and opinion.

To this disease some have conceded an independent existence, affecting the intestines chiefly, and there having its chief localization. Others have regarded it as a specific blood disease, in this view putting it into the category with typhus and cholera. In this view the localized affection of the intestines is only a result of this precedent fact. Others, as *Williams*, regard it as only one of the fruits of marsh malaria, and so in its origin they regard it as of the family which embraces the intermittent and remittent fevers, and their cognates. *Röderer* and *Wagler* take this view, while *Eisenmann* and *Canstatt* deny its independent existence, and consider it only as the localization of various pathological processes in the intestines, such as scurvy, typhus, rheumatism, and cholera. *Cœlius Aurelianus*, and *Stoll* regarded it as an internal rheumatism with ulceration; *Huxham* and *Broussais* as a simple inflammation of the colon; *Cullen* as a constriction of the intestines constituting an obstruction to the passage of its fecal contents, while *Zimmermann* and *Annesley* allege its cause to be found in a change in the character of the biliary secretion; *Johnson* and *Martin* attribute it to changed bile and cutaneous activity. With many its origin is only miasmatic, while many others hold it to be only from contagion, while there are still others who hold its *origin* to be malarious and its *extension* to be the result of contagion.

The great variety of opinions which have prevailed on this subject with the best minds of the profession have more than a historical interest to commend them to our attention. They are confidently presented as the strongest witnesses to the erroneous ideas of the nature of disease in general which have prevailed with a uniform continuance in the antique school of the profession from its origin to this day. However diverse these opinions, there is no one of them which has not something of fact on which it has been based. The second difficulty has been that observers have each confined their attention chiefly to such facts as favored their individual opinions, having no eyes for others, or giving them no place in the view from which their judgment was made up. With this partial view of the facts any philosophy of the disease was possible, and all were about equally plausible and worthless.

It is only when disease is recognized as a *state*, affecting the vital condition of the whole man, and not as a thing, localized in some isolated spot or organ, that a philosophy of individual forms of it which will bear scrutiny in the light of facts and be found equally applicable to all its examples can be possible. In this matter of the nature of disease in general the whole has been ordered and fixed by a power above all appeal, and nothing has been left to the disposal of those who make its cure their business but to see things as they *are*, and deal with them accordingly. Dogmatism here is of no importance, no matter how high the authority from which it comes. It is ever a cheap method in science. Here it is worthless and contemptible. To this failure to recognize the actual and fixed nature of disease in general, and to this partial observation of facts by those who have been the successive teachers of successive generations, is to be ascribed the ever-shifting and multitudinous theories which constitute so large a portion of the history of practical medicine for three thousand years. Teachers and writers before the time of Hahnemann failed to recognize the truth that Almighty power had established all the facts and relations of the case before their day, and that they have neither the power nor the calling to change them in the least particular. That their whole duty was to see, first, what God had done, and, second, to accept this and deal with it according to the requirements of the law He had enacted for the government and necessities of the case—that in this is comprised the whole sum of the physicians' practical duties. In this view, let us see what are the facts and the duties so established in the matter of dysentery.

And the first fact which we recognize is that this, like all other diseases, is a general fact, pervading the whole individual man, reaching to and affecting all the functions of his bodily organs, no one of them being left untouched. There is no vital action in the organism which is not changed. In this we only meet the fact equally true of all other diseases, and which, even in it and them, declares in language from the Divine power, the language of facts, that there is in the world no such thing as a *merely local disease*. Hence the views of *Huxham*, *Broussais*, and *Cullen*, and of all who, like them, have limited the action of the disease to the localized affection of the great intestine, are completely negatived at the very outset. They never have shown, and it never can be shown, that the inflammation and ulceration of this part is more an essential part of dysentery than is the loss of muscular force, the changed morale of the patient, or the universal change in the functions of the various secreting organs. Those who give it the general character of a disease of the blood are equally partial and faulty in their views, as are also those who limit its nature to change in one or more of the important secretions of the bodily organs. These certainly are affected, as these writers declare, but so are all the other secretions, and there has been no good reason given for making those of the liver and skin of such pre-eminent importance as to exclude the others from consideration when the verdict as to the general character of the disease is to be made up. There is as good reason for excluding these, to which such prominence has been given, as any one of those which have been so completely ignored in the presentation of this partial view, or, as for that, of any of the other general or local facts which have here been so unwarrantably omitted.

The second fact which arrests our attention is that, as a central point in this general affection, there is a group of phenomena which gives character to it among diseases, and without which no one of them is ever called dysentery. This is the group which characterizes the genus and gives it its place in the circle of those families of morbid processes which constitute the sum total of human diseases. In the practical relations of facts, this group has shown its chief significance and importance when it has decided that it is dysentery with which we have to do. Hence it is only the defining, or generic, group of facts or symptoms of the case. We say facts or symptoms, because all facts are symptoms, and all symptoms are facts—the terms are strictly interchangeable. So that when we, as a school medical practice, are accused in our practical consideration of diseases of dealing only with its symptoms, we accept the accusation as a truth. We deal as we profess, with its *facts*, and with nothing else. This has been by ignorance cast at us as a reproach. We accept it as an honor. In return, we only inquire of the opposer what it may be with which he deals, seeing he is so dissatisfied with *facts*.

This central group has its origin in a localization, not of *the disease*, but of one of its elements, or of one of the processes of which it is composed. This localization is in the large intestine. The process is an inflammation of that organ. The group of symptoms there originating is made up of *frequent and for the most part small discharges of blood or of bloody mucus from the rectum, with pain, tenesmus, and fever*. This has often been regarded as expressing the whole of the disease. It is only its generic or defining group of symptoms. In our practical endeavors to cure, this group has a much less important place than other and far less obtrusive facts. It simply determines the diagnosis, and then leaves the prescriber where he was before as to all knowledge of means for a cure. These are discovered chiefly by a careful consideration of the third fact which is present to our observation, viz.: that there is a peripheral group of symptoms gathered around this central and localized one which declares, not that we are dealing with *dysentery*, which has already been decided, but which goes beyond this, and declares the *kind* of dysentery which is before us. The importance of this peripheral group does not cease here. It extends far beyond, it being the group which contains the indices which point to the curative agencies, through the law of similars, on which we have learned we can safely depend.

It is the group to which we are to find the simillimum in the effects of some member of the materia medica on the living organism, as recorded in its pathogenesis, the law of cure demanding the similarity of these two classes of facts, of the drug and the disease. It is made up of far more numerous elements than the central, defining group, embracing, as it does, all those facts of any case not essential to constitute it a member of its class, but which belong to it as an individual member of that class. In other words, the group embraces all the specific symptoms of the case, while it includes none which are generic. As we have remarked, many of these are gathered around the generic group, and are found as concomitants of its members, or as circumstances or conditions by which they are excited, aggravated, or alleviated. To these are added those modifications of the functions of other organs which ever make up a very important part of the case, and are so ever varying in their character or circumstances as to constitute a large part of the elements which compose the characteristics of the case, which are our chief guides in the selection of the specific remedy. Let us look first at those elements of this peripheral or specific group which attach to and give character to those of the generic, viz.: the evacuations, the pain, the tenesmus, and the fever.

The discharges vary in their character. They may be at first pus mixed with mucus, or mucus and blood, or blood only. Later in the attack the pus is absent. The mucus may be yellow, green (light or dark), or brown. The blood may be bright or dark colored, mixed with other matters, or in separation from them. It may be fluid or coagulated, in streaks or specks. The voided mass may be odorless or offensive. The offensive odors are various in character. It may be like that of spoiled eggs or of putrid flesh, or it may be of a penetrating, disgusting, indescribable character; or the discharges may be watery, ichorous, or purulent, brown, green, gray, yellow, mottled, blackish, sticky, tarlike, or mixed with yellowish flakes or patches of membranous exudation. The evacuations, though for the most part small in quantity and of frequent occurrence, vary much in different cases in both particulars.

The concomitant symptoms of the evacuations are very different in different cases. There may be before the evacuations thirst, nausea, vomiting, anxiety, restlessness of body and mind, faintness, perspiration, partial or general, which may be cold or hot. There may be any of these symptoms present with the evacuation or after it.

Disposition to evacuation may be excited by the ingestion of the smallest quantity of food or drink, and also by any, even the slightest, movements of the body. The greatest sense of exhaustion may attend or follow the discharges. These may also be preceded, attended, or followed by shudderings, chill, heat, or sweating.

The pain is very various in its character, as cutting, pinching, burning, excruciating, bruised, constricting. It varies in location, as in the hypogastrium or the region of the navel. It may extend from the intestines to other near or remote parts, as the urinary bladder, the loins, the sacral region, or the thighs. It may be present in its greatest severity before and during the evacuation, and cease, for the time, immediately after, or it may be continued after with equal or nearly equal severity. It may be relieved by particular positions of the body or limbs, by external warmth, and in some cases by a moderate external pressure. It may be renewed or intensified by food and drinks of whatever kinds.

The tenesmus is present in different degrees of severity, with accompanying pains in the anus of different character. There may be a sense of this part being torn out or constricted, or there may be burning, smarting, cutting, stabbing, shooting, or throbbing in the part accompanying the tenesmus. This symptom may be found to cease with the accomplishment of the evacuation or to continue after it.

The fever is also various in its intensity and accompaniments. In some cases it is developed in a slight chill or shuddering, followed by a similar slight reaction of heat of the surface and acceleration of the pulse, which disappears after the first day or two of the attack. At other times these elements are more positive and persistent in their character. In other cases the elements of fever are developed later in the case, and are indicative of and spring from important changes in the condition of the part where the diseased process is more especially localized. The two forms of fever are quite different in importance and significance. The mildness or severity of the first is no measure of the danger of the patient; neither is its cessation evidence of convalescence, or even that the worst of the attack has passed. On the contrary, the second is always indicative of grave and important changes, and its cessation may unhesitatingly be regarded as a favorable indication in the case.

The elements of the fever in this, as in other diseases, have their particular characteristics. The chill may be a general sense of coldness, with shuddering, varying in its duration in different cases, or there may be only slight, creeping chilliness, confined to the back or limbs, or it may be in the form of chilliness of the upper or lower extremities or of either side. The heat may be general or partial, extreme or moderate in degree, with great or slight restlessness, and with thirst intense, slight, or not at all present. The perspiration, if there be any, may vary in its character, and also be general or partial, hot, warm, or cool.

The general symptoms also belong to this group, such as debility exhaustion, emaciation, faintness or fainting, either in connection with the evacuations or independent of them; coldness of surface, which is dry or covered with perspiration; heat of surface, dry or sweating; color of general surface, as pale, red, or bluish; painful sensibility of the general surface to touch or pressure; sensation of being generally bruised; cramps in the limbs; general restlessness, with or without tossing about in the bed; sense of paralytic weakness in the limbs; sensibility to the open air, even though it be warm; sensibility to external cold; the position in bed.

So do also the functional symptoms of other organs than that more especially affected by the localized process in the large intestine. Of the skin there is to be noted temperature, perspiration, its general or local characters, its smell, if any, and the stain it leaves on the clothing, if any; the expression of countenance; the color of the face, as pale, red, or bluish. Is the face turgid and full, as if bloated, or shrunken, and the features sharpened? The state of the lips, are they pale or red, dry and cracked, or smooth? Is the mouth dry, or covered with mucus? The tongue dry or moist, clean or coated? Modifications of taste are also to be noted; as is also the odor of the breath, if this be offensive; apthæ in the mouth; loss of appetite, thirst, or repugnance to or desire for particular forms of food and drink; difficulty of swallowing and hiccough; nausea, and if there be vomiting, the character of the ejected substances; distention of the abdomen, with or without sensibility to external pressure; if sensitive, the quality of the pain produced, as of cutting, excoriation, or bruise; prolapsus of the rectum with the evacuations; tenesmus of the urinary bladder; the character of the urine voided; sleeplessness; coma; anxiety; delirium; the state of the pulse, and the like.

These are the phenomena which gather around the central, localized process of the disease, and declare its specific character; and, in so doing, point at the same time to the specific remedy which cures. Hence it is these, in our practical duties, that chiefly engage our attention. In the two aspects in which, as practical physicians, we are compelled to view diseases, first as facts in science, second as objects of our practical duties, this peripheral group of symptoms belongs by eminent importance to the latter. Though its members belong to the disease, and are as really integral parts of it as are the generic symptoms in its scientific existence and relations, still they have their highest importance in their office of guides to the selection of specific remedies. They are seldom or never all present in any one case; oftener there are but few of them, but these few are no less the guides to our choice because they are few. Whether few or many, they are our only guides to a safe and sure practice. The enlightened and conscientious physician will give no less heed to them in this case, and never, for this reason, turn from them to any, whatever, of routine resort, because Doctor this or that declares that he "cures all his cases by it." By this he simply proves that he does not know what a *cure* really is.

A moderate intensity of the pain and tenesmus and evacuations of moderate frequency and quantity, which are not very offensive and contain but little blood, are favorable, while the reverse of these are unfavorable and in the ratio of the intensity in which this reverse condition is present. Other unfavorable elements are a long continuance of the disease, which is threatening to pass into a chronic form; relapses are more dangerous than original attacks; symptoms of collapse; great prostration of the vital forces; symptoms of peritonitis and of perforation of the intestine, chills, a falling in of the abdominal parieties, with loss of their natural elasticity; involuntary evacuations, erysipelas, pyæmia, violent vomiting, with choleraic symptoms; rapid emaciation, dropsical affections, protracted hiccough, delirium, convulsions, and paralysis.

Treatment.—When we pass from the consideration of dysentery as a fact in medical science, to treat of it as a fact to be cured, the first question we have to answer is, How shall we be able to grasp with certainty the means best adapted to the accomplishment of this end? And if, just here, the law of similars presents itself, and claims our confidence as a sure guide in the selection of the requisite means, we reply with the next question, What does this law of similars require? Or, in other words, what is the simillimum which it requires us to discover to be like? What are the facts which it is to resemble, the likeness to which constitutes it the curative of the case, under the authority of law? To be able to answer this question with clearness and certainty when it arises, as it must, at the outset of our practical consideration of our subject, we have already presented a somewhat extended statement of those facts in two classes, viz., the generic and specific symptoms of the case. Similarity to these alone answers the demands of the law. Not to the mass of these, as we have presented them as a picture of the general subject, but to such of them as may have presented themselves in the particular case we are about to treat. To these we are to find the simillimum and we have found the cure. And this is what we are to do in each succeeding case. It is to be understood, now and ever, that there is no such thing as a cure for this or any other disease in any single drug; though it need not be forgotten that there are not wanting those who say they cure *all* their cases with one remedy—*Merc. corr.*, for example. They are probably honest in their statements, and mean to be truthful. The difficulty with this class of practitioners is not that they are, as a rule, not honest, but that they have an imperfect idea of what constitutes a cure and a very limited experience of that which they fail so completely to comprehend. A cure with this class of practitioners is one thing—a cure with those who really find the simillimum of each case, as it is treated, is quite another. In the one case the idea of *cure* is simply that of *recovery*—in the other it is the positive change of the destructive action of the vital forces, by the efficient agency of a medicinal force, to that conservative balance of action we call health. With the one, the idea of a cure is little more than a negative; with the other, it is a positive removal of an evil by the interposition of an efficient agency, in compliance with the demands of a divinely appointed law. Recovery may be possible where no means are employed for the attainment of this end, or even where the means employed have been wholly wrong, and therefore, so far as they have been influential in the case, have been only hurtful. A cure results only from the right use of right means, according to the requirements of law, the whole tendency and influence of which, when so employed, is in the direction of health, and therefore only beneficial.

There is, sometimes, an experience in the treatment of this disease, when it is present as an epidemic, which at first glance would seem to negative the statement that it finds its cure in no one drug. There is such a repetition of the facts of each case in every other, in these circumstances, as to make it quite possible for a cure to be found in a single drug for the epidemic. The reason for this is in the fact that all the examples of the disease in the epidemic are the product of a common cause, the action of which on the organism is so little modified by local or individual circumstances that the result in each case is virtually a representative of that in every other. The resemblance of each case to each is so great that the simillimum to one is a simillimum to the whole. So that, though a single drug may be found which will cure most, or even all, the examples or an epidemic, it cures by virtue of the resemblance of its effects on the organism to the symptoms of each or all the cases of the epidemic, and not at all because the case in hand is a dysentery. The cure is general only because of the uniformity of the symptomatology of the cases, and the application of the drug in each succeeding case is as much an individual compliance with the demand of the law for an individual likeness of the drug and the symptoms of the case, as if there had been but this one case to be treated. It will be apparent then, at once, that this experience in epidemics of dysentery, or of any other disease, is only an apparent, and not a real negation of our denial to any one drug of the right to be regarded as the curative of the genus dysentery.

And then, it is not to be forgotten that when the cure for the epidemic which now prevails has been found, and it has cured each of one hundred cases without a failure, we have done nothing toward finding cures for subsequent epidemics of the same disease beyond the discovery of the method of finding cures for epidemics in general, if, indeed, we have found this. It is certain that the drug which cured every case of the epidemic twelve years ago, has not since sustained the same relation to any succeeding one, and has rarely cured even in a single case. It will be in place again only when epidemics or cases present a symptomatology like to its effects on the organism, as that did in which it was so uniformly curative.

In proceeding to treat a case of this disease, *i. e.*, to find the specific remedy for its cure, the first caution we should regard is not to allow ourselves to be too much occupied by the central or generic group of symptoms. There is no small difficulty in avoiding this, for here are present the chief sufferings of the patient, and these wholly occupy his attention and that of his friends, and it is these they will constantly thrust before the prescriber, all else to them being of comparatively small moment. For this and other reasons we are to look elsewhere for the controlling facts which decide the choice of curative means. We are to look elsewhere for these facts, because, as we have already intimidated, they are not to be found in the central group, and because if we do not find them elsewhere, no one else will find them for us. If others have knowledge of their existence, they have no suspicion of their importance, and they are sure to be passed by as of little consequence. If we do not seek them out and give them their due place in our practical decisions, we treat our case in darkness, and our patient is likely to escape a *cure*, and take his chances for a *recovery*, whatever these may happen to be. We are not only to look outside of this central group for our guiding symptoms, but are to be satisfied with nothing short of gathering every fact from every other quarter, and of whatever kind, and bring them all into our view, and hold them there till we have made our selection of our remedy. In this duty we shall not be embarrassed by the presence of *all* the many peripheral symptoms we have named in a single case. Probably there will be but few of them. Few or many, here are our guides when we have them once before us.

But the difficulty as to symptoms is not ended when we have progressed only thus far. However few or many of these are found in the case, they are not all of equal importance as indices of its true curative.

We are now to further examine all these, and make selection of those which are most authoritative in the decision of our choice of a remedy. In making this further elimination, we are to be guided by the same general principle which excludes so largely from the process of prescribing those symptoms which belong to all the members of the class. There are, in the peripheral group, those members which are oftener repeated in the progress of successive cases than others. As these are common to many cases, so analogues of them may be found in the pathogenesis of several or many drugs. Therefore there is in these little or nothing which enables the prescriber to decide which of these drugs he is to select for a given case. And just in proportion as the commonness of these symptoms brings them near in character to the generic group, in that proportion they pass out from among those elements of the case he is chiefly to consider. When these have been generally set aside, the first great difficulty in the way of finding a curative is removed. There will remain, then, a group of symptoms, composed of members more or less numerous, which belong to the case as an individual member of the family or class. They are the features by which it is known in the family as a distinct member, and which distinguish it from the other members. In other words, they are the elements of the case which give to it its individuality, and are what we mean when we speak of *characteristic* symptoms, because they, and only they, declare its real character in its relation to its specific curative agent. The office of the prescriber, as such, is little more than finding that drug which in its record of proving on the healthy has been found to produce symptoms like those so found to be characteristic of the case before him. As those symptoms of the case are not common, but peculiar to it, so the similar symptoms of the drug will be found to be peculiar to it, and are what we mean when we speak of the characteristic symptoms of the drug. It is in the resemblance between these two classes of characteristics that the divinely ordained law of cure has its existence. And in no one arrangement in nature is the wisdom and benevolence of the Lord given more conspicuousness than in this. If the diseases of men were to be cured by agencies from without the organism, and the arrangement for this were to be made of such a general character as, when discovered, it should be found to be of the nature and force of *law*, so that the curing process should be one of comparative certainty, and in no case be left to the contingencies of chance, then it is submitted that this is the only possible management that could secure these ends. If this law of curative relationship had been established between those elements of the disease and drug which are common to many members of each class, how could we ever be certain which of the many drugs, characterized equally by similarity to the general elements of the disease, would be its true curative? In this case, it is clear, certainty would be impossible. And by parity of reasoning, it follows that certainty is possible, and not a very great difficulty, if it be found that the law appointed by which these diseases are to be cured has been ordained in the similarity of those elements of the disease and drug which are peculiar to each, *i. e.*, of their characteristics. Careful observation and experience have abundantly taught us that it is just here, where intelligent minds could alone have expected to find it, that the law of cure exists. And more than this: They teach that the application of means to the cure of disease, selected and used in compliance with the requirements of this law, as stated above, is followed by a uniformity of success so great as to warrant its expectation with an assurance little short of certainty.

It only remains, in our consideration of this subject, to show how these general principles are applied to the treatment of particular cases. The object being to find the drug which cures, how are we to proceed, under their guidance, to its discovery? It must be obvious, at the first glance, that the subject, from its nature, admits of no such exposition as will show what the remedy must be for each successive case. The most that can be done is to show how that remedy is to be found. Of course, it cannot be discovered before the case arises which is to be cured, and therefore only a general *a priori* consideration of the details of treatment is possible. In our endeavors to present the true method of proceeding, we shall give representative cases of the disease as allied to particular remedies.

If the case be related to *Aloes*, we shall find, besides the generic symptoms of the disease, some of the following: *Fainting while at stool*—very characteristic of this drug; frequent stools of *bloody water*, the *tenesmus is very violent; hunger* during the stool;

*shooting* and *boring* pains in the region of the navel, *increased by pressure;* the *lower part* of the abdomen is *swollen and sensitive to pressure;* the distention and movements in the abdomen are more in the *left side* and along the track of the colon, *increased after food;* great repugnance to free air, which, notwithstanding, ameliorates the sufferings; *cutting* and *pinching* pains in the *rectum* and *loins;* heaviness, weariness, and numbness in the thighs. With these symptoms there need be no hesitation as to the choice of *Aloes.* Many of these symptoms are found with no other drug so far as we know.

If related to *Arnica,* there will be some of the following: Constant sense of fullness and satiety in the stomach, with nausea; putrid and slimy taste in the mouth; taste and eructations like spoiled eggs; bitter and sour eructations; putrid smell of the breath; loud rumbling in the bowels, as if empty; stools of *blood* and *pus;* offensive flatus like bad eggs; swallowing hindered by a sensation of nausea; repugnance to animal food and broths; wishes to drink constantly, but does not know what, all drinks are alike offensive; tenesmus of the neck of the bladder; fruitless urgency to urinate (*Merc. cor.*); bruised pain in the back; painfully increased sensibility of the whole surface of the body; perspiration smells sour. It will be noted how different these symptoms are from those of *Aloes.* There can be no difficulty in deciding between the two in any case. There is just as little between both these and the next we note, which is

*Arsenicum.*—Here we have sensation as if the *abdomen would burst before* the stool; sensation of *contraction,* just *above* the *anus at* the stool; burning in the rectum and trembling in all the limbs *after* the stool; heart-beating and distention of the abdomen *after* the stool; tenesmus, with burning in the rectum and anus (*Caps.*); great exhaustion *after each stool;* stools smelling like old foul ulcers; greenish urine; pains relieved by external heat; bluish tongue; great restlessness and tossing about the bed; face sunken, pale, and the features distorted; perspiration *sticky;* petechial, milliary, and nettle-rash eruptions; cold, dry skin alternates with cold perspiration; pain relieved *after* each evacuation. These are quite characteristic symptoms of this drug, and are easily distinguished from those of almost all others. Of these are to be more especially noted the concomitants *before, during,* and *after* the stool, the great restlessness and the exhaustion after the stool, as well as the character of the perspiration.

*Belladonna* is more likely to be appropriate in the early stage of the disease and when the inflammation extends to the serous tissues of the intestines. This is shown by the presence of symptoms which characterize that condition, such as *deep*-seated soreness of the abdomen when pressed on; *hard*, quick pulse; hot, dry skin, with evident congestion of this tissue. In the initiation of the case there may be chills, excited by every motion (*Nux v.*), or frequent alternations of chilliness and flashes of heat, both being transient and in rather quick succession. Other drugs have the sensibility of the abdomen to pressure, as, for example, *Hyos.*, *Nux v.*, *Puls.*, *Sulph.*, and some others. It will be necessary, therefore, to note that the character of the sensibility with *Bell.* is that of excoriation, as if *all were raw* within, and also the febrile symptoms, including the pulse. If these are as we have just given them, there is the strongest reason for the selection of this drug. It is the more certainly indicated if there be a constant pressing to the anus and genitals; if the pains are more in the left side, and are aggravated by bending the body to that side; if there be pains of a constricting character, relieved by bending forward; painless inability to swallow; sensation of dryness in the mouth while the tongue is moist; violent delirium.

If the case call for *Cantharis* with other symptoms, there will be burning in the *anus* like *fire, after* the stool; dryness of the lips, and thirst during the pain; loss of epithelium from the lips, tongue, and palate; vesicles and apthous ulcers in the mouth and throat. There may be also this peculiarity of the evacuations—like *scrapings* from the mucous surface of the intestines, *streaked* with blood.

*Capsicum* has thirst after every evacuation and shuddering after every drinking; stool after each drinking; taste like putrid water; tenesmus of the bladder (*Merc. cor.*); pains aggravated by currents of air, though warm; coldness of the body without shuddering; drawing pains in the back, which, with the tenesmus, are continued *after the stool;* thin, adhesive slime mixed with black blood, with twisting pains about the navel. This is one of the most important remedies in dysentery, and is nearly allied to *Nux v.* and *Merc.* We shall give the distinctions by and by.

*Colchicum* has cramps in the calves of the legs, prolapsus of the anus with the evacuation, constriction of the œsophagus, great swelling of the lower part of the abdomen, frequent shudderings down the back. It is said to be curative when the stools are more mucus than blood, and after sublimate has failed in such cases.

*Colocynth.*—If there be fruitless efforts to vomit, weakness, paleness, and prostration *after* the stool (*Ars.*); burning pain along the sacral region. The pains are cutting and squeezing and extremely severe, often accompanied by retching and bending the body forward, and are partially relieved by external pressure. With the severe pain there are shudderings on the cheeks, which seem to come from the abdomen, with relief of the pain. The pains are such as characterize neuralgia rather than inflammation of the intestines; they are relieved by *Coffea*, and the relief is followed by immediate disposition to stool. Cramps and cramp-like contractions of the muscles of the body, cold hands, with warm feet. It is oftener appropriate in the early than later period of the attack. There is a senseless practice with some of giving "*Colocynth for the pain*" and other drugs for supposed alliance to other elements of the attack, and these in alternation, according to the fancy of the prescriber, and not in accordance with any known law of nature. All such proceedings are the offspring of imperfect intelligence and can have no countenance from the instructed practitioner. There is another habit of some, who give this drug in all their cases of dysentery from routine or habit. Against both these we protest, as neither in accordance with the requirements of the homœopathic law nor in any way beneficial in practice. The true homœopathic application of this drug in this disease is rather restricted to the few cases than extended to the many. As benefit can only come from its use when it is truly homœopathic, a careful study of its symptoms is urged, and that it be only given where these sanction its use according to law. The other course is only at the expense of the sufferings of the patient, of precious time, and, it may be, of his safety. The possible benefit is not such as to warrant these risks.

*Cuprum metallicum* if there be severe *retching* with the stool; cramps in the fingers and toes; sweet, ropy saliva; paralytic sensation in the arms and feet; slimy mouth; sweet taste in the mouth; all food tastes like clear water; hiccough; retching, with cramp-like pains in the abdomen; downward pressure in the hypogastrium like a stone; distention of the lower part of the abdomen; hardness of the abdomen with great sensibility to pressure; severe cramps in the abdomen and upper and lower extremities; comatose sleep after vomiting.

*Mercury* has cuttings in the lower part of the abdomen at night. The abdomen is externally cold to touch. Cutting stitch in the lower part of the abdomen, from right to left, and aggravated by walking; fecal taste in the mouth; putrid taste in the throat; salt saliva; nausea with vertigo; obscured vision, and flashes of heat; offensive perspiration. The pains are increased *before* and *during* the stool, with violent tenesmus. The pains are rather increased than diminished *after* the stool, and sometimes then extend to the back. The tenesmus as well as the pain is continued *after* the stool. *During* the stool hot sweat on the forehead, which soon becomes cold and sticky. Drawing pains in the lower extremities, which impel to frequent changes of position; dry, cracked lips. The discharges are excoriating.

*Mercurius cor.*—This drug has been more used, empirically, by some physicians of our school in the treatment of dysentery than any other. We say empirically, because there is no such proving of it as will direct its use otherwise. In the brief proving given by Hahnemann, there is a single statement of a group of symptoms which belong to the generic symptoms of this disease, and this is all. Still, some have claimed to cure every case of dysentery presented to their practice by this drug alone, in a time and manner quite satisfactory to themselves; others have been less successful with it, and some have realized little of good from its use. If it is difficult to reconcile these discrepancies in practice, it is not permitted us to doubt their actual existence. For many years in my own practice I gave the drug very many times, in different preparations and different potencies, without in any case being able to see that any good resulted. The same uniform want of success attended its use in the practice of several of my friends. It became certain to my mind that the clew to its right place in the treatment of the disease was wanting. Specific symptoms in the materia medica were wholly wanting. That the drug had some relation to the disease there could hardly be a doubt. But what, and how was it to be ascertained? In *vols.* I, II, and III of *Frank's Magazine*, cases of poisoning are reported from which same symptoms have been obtained, which possibly may have some value in aiding us to answer the question. It is not to be forgotten that while symptoms of real value may be derived from such cases, as a rule they occupy the lowest place in the modes of proving drugs. The disturbances in such cases are violent, brief, and destructive, and often before there has been time for the development of those specific symptoms most characteristic of the drug, and therefore of the greatest value in practice, life itself is destroyed, and only the more common symptoms of poisoning are observed, and the whole is of little practical value. The following symptoms have been extracted from these cases and may be worth our attention as undoubtedly having been results of the action of the drug:

Cold face and hands, with small and feeble pulse. Lips dark red and swollen. All the pains, but especially those of the rectum, are aggravated by motion. Pulse small, hard, and frequent. Coma. Cramps in arms, hands, and fingers, legs, feet, and toes. Faintings. Weakness and shuddering in the limbs. The limbs as if bruised and trembling. Great anxiety and palpitation of the heart. Wandering shiverings. Sensation of coldness, pale face, and slight nausea. Coldness of the lower part of the abdomen. Abdomen tense, hard, and sensitive to pressure, especially about the navel. Obstinate sleeplessness. Dysphagia. Astringent, metallic taste in the mouth. Great prostration. Great prostration after the vomiting of food. Hiccough. Frequent eructations. Painful pinchings in the stomach. Spasmodic, watery vomiting, without previous nausea. Severe shooting pains in the stomach and liver, with vomiting of bile. Drinks are immediately vomited, with great effort, mixed with tenacious, stringy mucus. Severe pains in the rectum which continue after the discharges. The fruitless urgency to stool increases the pains. Pain extends from the navel to the back. Distention of the abdomen, with borborygmus. Evacuations very offensive. Suppression of the secretion of the urine. Retention of urine.

In addition to the above symptoms and in harmony especially with the two last, it may be remembered that Prof. Carroll Dunham some years since, after disappointments in the use of sublimate similar to my own, found it promptly efficacious in cases complicated with urinary tenesmus. My own experience has since confirmed his judgment that the particular province of sublimate in dysentery is in the treatment of cases with this peculiar complication. It may have other specific relations to this disease which remain for some other Dunham yet to discover. I have found it curative in cases with *suppressed urinary secretion.*

*Nux vom.* has small, frequent evacuations, with violent tenesmus; pressing pains in the loins and upper part of the sacral region, with

### CHAPTER I.

### DYSENTERY.

### Its History, Pathology, and Treatment.

Medical writers from the time of Hippocrates have treated of dysentery, and the variety of opinions they have entertained is but little less than the number of writers by whom the subject has been discussed. Three thousand years have not sufficed to settle the question for all whether it be really a disease of independent existence, or whether it be only an adjunct of some other more general form of evil. Those who have regarded it as an independent disease have had no agreement of views as to its true nature, while those who have contended for its derived status have failed equally to agree as to the origin to be assigned to it and the nosological relations in which it ought to be placed. From Hippocrates and Celsus, Aretæus and Galen, through the long list of those of the ancient and middle ages, as well as through that of the modern, down to the latest and best of the writers of our own day, nothing is established but a great diversity of opinion, though nearly all have tried their best to remove difficulties and differences. But even now, in works of the latest date and most complete character, the subject is discussed as an unsettled question, and *Bamberger* seems to come no nearer to its adjustment than Hippocrates or Galen. He says upon the nature and general nosological relations of this fatal disease there are not only the most various opinions expressed, but that these are in many instances no less than the exact opposites of each other. He ascribes this well-known fact in part to the different pathological systems and opinions advocated by different writers, and in part to the difference in times and circumstances in which the disease has been developed. The whole difficulty has grown out of an unwillingness on the part of these writers to concede to the disease an existence, and a nature outside and independent of their systems and opinions which by its visible phenomena it has never failed to assert. While this was clearly seen as to the systems and opinions of precedent writers, singularly enough, each was as blind to the fact in its application to his own as were all his predecessors. The profession seems to have been incapable of conceiving of disease outside of this circle of system, relationship and opinion.

To this disease some have conceded an independent existence, affecting the intestines chiefly, and there having its chief localization. Others have regarded it as a specific blood disease, in this view putting it into the category with typhus and cholera. In this view the localized affection of the intestines is only a result of this precedent fact. Others, as *Williams*, regard it as only one of the fruits of marsh malaria, and so in its origin they regard it as of the family which embraces the intermittent and remittent fevers, and their cognates. *Röderer* and *Wagler* take this view, while *Eisenmann* and *Canstatt* deny its independent existence, and consider it only as the localization of various pathological processes in the intestines, such as scurvy, typhus, rheumatism, and cholera. *Cœlius Aurelianus*, and *Stoll* regarded it as an internal rheumatism with ulceration; *Huxham* and *Broussais* as a simple inflammation of the colon; *Cullen* as a constriction of the intestines constituting an obstruction to the passage of its fecal contents, while *Zimmermann* and *Annesley* allege its cause to be found in a change in the character of the biliary secretion; *Johnson* and *Martin* attribute it to changed bile and cutaneous activity. With many its origin is only miasmatic, while many others hold it to be only from contagion, while there are still others who hold its *origin* to be malarious and its *extension* to be the result of contagion.

The great variety of opinions which have prevailed on this subject with the best minds of the profession have more than a historical interest to commend them to our attention. They are confidently presented as the strongest witnesses to the erroneous ideas of the nature of disease in general which have prevailed with a uniform continuance in the antique school of the profession from its origin to this day. However diverse these opinions, there is no one of them which has not something of fact on which it has been based.

The second difficulty has been that observers have each confined their attention chiefly to such facts as favored their individual opinions, having no eyes for others, or giving them no place in the view from which their judgment was made up. With this partial view of the facts any philosophy of the disease was possible, and all were about equally plausible and worthless.

It is only when disease is recognized as a *state*, affecting the vital condition of the whole man, and not as a thing, localized in some isolated spot or organ, that a philosophy of individual forms of it which will bear scrutiny in the light of facts and be found equally applicable to all its examples can be possible. In this matter of the nature of disease in general the whole has been ordered and fixed by a power above all appeal, and nothing has been left to the disposal of those who make its cure their business but to see things as they *are*, and deal with them accordingly. Dogmatism here is of no importance, no matter how high the authority from which it comes. It is ever a cheap method in science. Here it is worthless and contemptible. To this failure to recognize the actual and fixed nature of disease in general, and to this partial observation of facts by those who have been the successive teachers of successive generations, is to be ascribed the ever-shifting and multitudinous theories which constitute so large a portion of the history of practical medicine for three thousand years. Teachers and writers before the time of Hahnemann failed to recognize the truth that Almighty power had established all the facts and relations of the case before their day, and that they have neither the power nor the calling to change them in the least particular. That their whole duty was to see, first, what God had done, and, second, to accept this and deal with it according to the requirements of the law He had enacted for the government and necessities of the case—that in this is comprised the whole sum of the physicians' practical duties. In this view, let us see what are the facts and the duties so established in the matter of dysentery.

And the first fact which we recognize is that this, like all other diseases, is a general fact, pervading the whole individual man, reaching to and affecting all the functions of his bodily organs, no one of them being left untouched. There is no vital action in the organism which is not changed. In this we only meet the fact equally true of all other diseases, and which, even in it and them, declares in language from the Divine power, the language of facts, that there is in the world no such thing as a *merely local disease.* Hence the views of *Huxham*, *Broussais*, and *Cullen*, and of all who, like them, have limited the action of the disease to the localized affection of the great intestine, are completely negatived at the very outset. They never have shown, and it never can be shown, that the inflammation and ulceration of this part is more an essential part of dysentery than is the loss of muscular force, the changed morale of the patient, or the universal change in the functions of the various secreting organs. Those who give it the general character of a disease of the blood are equally partial and faulty in their views, as are also those who limit its nature to change in one or more of the important secretions of the bodily organs. These certainly are affected, as these writers declare, but so are all the other secretions, and there has been no good reason given for making those of the liver and skin of such pre-eminent importance as to exclude the others from consideration when the verdict as to the general character of the disease is to be made up. There is as good reason for excluding these, to which such prominence has been given, as any one of those which have been so completely ignored in the presentation of this partial view, or, as for that, of any of the other general or local facts which have here been so unwarrantably omitted.

The second fact which arrests our attention is that, as a central point in this general affection, there is a group of phenomena which gives character to it among diseases, and without which no one of them is ever called dysentery. This is the group which characterizes the genus and gives it its place in the circle of those families of morbid processes which constitute the sum total of human diseases. In the practical relations of facts, this group has shown its chief significance and importance when it has decided that it is dysentery with which we have to do. Hence it is only the defining, or generic, group of facts or symptoms of the case. We say facts or symptoms, because all facts are symptoms, and all symptoms are facts—the terms are strictly interchangeable. So that when we, as a school of medical practice, are accused in our practical consideration of diseases of dealing only with its symptoms, we accept the accusation as a truth. We deal as we profess, with its *facts*, and with nothing else. This has been by ignorance cast at us as a reproach. We accept it as an honor. In return, we only inquire of the opposer what it may be with which he deals, seeing he is so dissatisfied with *facts*.

This central group has its origin in a localization, not of *the disease*, but of one of its elements, or of one of the processes of which it is composed. This localization is in the large intestine. The process is an inflammation of that organ. The group of symptoms there originating is made up of *frequent and for the most part small discharges of blood or of bloody mucus from the rectum, with pain, tenesmus, and fever.* This has often been regarded as expressing the whole of the disease. It is only its generic or defining group of symptoms. In our practical endeavors to cure, this group has a much less important place than other and far less obtrusive facts. It simply determines the diagnosis, and then leaves the prescriber where he was before as to all knowledge of means for a cure. These are discovered chiefly by a careful consideration of the third fact which is present to our observation, viz.: that there is a peripheral group of symptoms gathered around this central and localized one which declares, not that we are dealing with *dysentery*, which has already been decided, but which goes beyond this, and declares the *kind* of dysentery which is before us. The importance of this peripheral group does not cease here. It extends far beyond, it being the group which contains the indices which point to the curative agencies, through the law of similars, on which we have learned we can safely depend.

It is the group to which we are to find the simillimum in the effects of some member of the materia medica on the living organism, as recorded in its pathogenesis, the law of cure demanding the similarity of these two classes of facts, of the drug and the disease. It is made up of far more numerous elements than the central, defining group, embracing, as it does, all those facts of any case not essential to constitute it a member of its class, but which belong to it as an individual member of that class. In other words, the group embraces all the specific symptoms of the case, while it includes none which are generic. As we have remarked, many of these are gathered around the generic group, and are found as concomitants of its members, or as circumstances or conditions by which they are excited, aggravated, or alleviated. To these are added those modifications of the functions of other organs which ever make up a very important part of the case, and are so ever varying in their character or circumstances as to constitute a large part of the elements which compose the characteristics of the case, which are our chief guides in the selection of the specific remedy. Let us look first at those elements of this peripheral or specific group which attach to and give character to those of the generic, viz.: the evacuations, the pain, the tenesmus, and the fever.

The discharges vary in their character. They may be at first pus mixed with mucus, or mucus and blood, or blood only. Later in the attack the pus is absent. The mucus may be yellow, green (light or dark), or brown. The blood may be bright or dark colored, mixed with other matters, or in separation from them. It may be fluid or coagulated, in streaks or specks. The voided mass may be odorless or offensive. The offensive odors are various in character. It may be like that of spoiled eggs or of putrid flesh, or it may be of a penetrating, disgusting, indescribable character; or the discharges may be watery, ichorous, or purulent, brown, green, gray, yellow, mottled, blackish, sticky, tarlike, or mixed with yellowish flakes or patches of membranous exudation. The evacuations, though for the most part small in quantity and of frequent occurrence, vary much in different cases in both particulars.

The concomitant symptoms of the evacuations are very different in different cases. There may be before the evacuations thirst, nausea, vomiting, anxiety, restlessness of body and mind, faintness, perspiration, partial or general, which may be cold or hot. There may be any of these symptoms present with the evacuation or after it.

Disposition to evacuation may be excited by the ingestion of the smallest quantity of food or drink, and also by any, even the slightest, movements of the body. The greatest sense of exhaustion may attend or follow the discharges. These may also be preceded, attended, or followed by shudderings, chill, heat, or sweating.

The pain is very various in its character, as cutting, pinching, burning, excruciating, bruised, constricting. It varies in location, as in the hypogastrium or the region of the navel. It may extend from the intestines to other near or remote parts, as the urinary bladder, the loins, the sacral region, or the thighs. It may be present in its greatest severity before and during the evacuation, and cease, for the time, immediately after, or it may be continued after with equal or nearly equal severity. It may be relieved by particular positions of the body or limbs, by external warmth, and in some cases by a moderate external pressure. It may be renewed or intensified by food and drinks of whatever kinds.

The tenesmus is present in different degrees of severity, with accompanying pains in the anus of different character. There may be a sense of this part being torn out or constricted, or there may be burning, smarting, cutting, stabbing, shooting, or throbbing in the part accompanying the tenesmus. This symptom may be found to cease with the accomplishment of the evacuation or to continue after it.

The fever is also various in its intensity and accompaniments. In some cases it is developed in a slight chill or shuddering, followed by a similar slight reaction of heat of the surface and acceleration of the pulse, which disappears after the first day or two of the attack. At other times these elements are more positive and persistent in their character. In other cases the elements of fever are developed later in the case, and are indicative of and spring from important changes in the condition of the part where the diseased process is more especially localized. The two forms of fever are quite different in importance and significance. The mildness or severity of the first is no measure of the danger of the patient; neither is its cessation evidence of convalescence, or even that the worst of the attack has passed. On the contrary, the second is always indicative of grave and important changes, and its cessation may unhesitatingly be regarded as a favorable indication in the case.

The elements of the fever in this, as in other diseases, have their particular characteristics. The chill may be a general sense of coldness, with shuddering, varying in its duration in different cases, or there may be only slight, creeping chilliness, confined to the back or limbs, or it may be in the form of chilliness of the upper or lower extremities or of either side. The heat may be general or partial, extreme or moderate in degree, with great or slight restlessness, and with thirst intense, slight, or not at all present. The perspiration, if there be any, may vary in its character, and also be general or partial, hot, warm, or cool.

The general symptoms also belong to this group, such as debility exhaustion, emaciation, faintness or fainting, either in connection with the evacuations or independent of them; coldness of surface, which is dry or covered with perspiration; heat of surface, dry or sweating; color of general surface, as pale, red, or bluish; painful sensibility of the general surface to touch or pressure; sensation of being generally bruised; cramps in the limbs; general restlessness, with or without tossing about in the bed; sense of paralytic weakness in the limbs; sensibility to the open air, even though it be warm; sensibility to external cold; the position in bed.

So do also the functional symptoms of other organs than that more especially affected by the localized process in the large intestine. Of the skin there is to be noted temperature, perspiration, its general or local characters, its smell, if any, and the stain it leaves on the clothing, if any; the expression of countenance; the color of the face, as pale, red, or bluish. Is the face turgid and full, as if bloated, or shrunken, and the features sharpened? The state of the lips, are they pale or red, dry and cracked, or smooth? Is the mouth dry, or covered with mucus? The tongue dry or moist, clean or coated? Modifications of taste are also tc be noted; as is also the odor of the breath, if this be offensive; apthæ in the mouth; loss of appetite, thirst, or repugnance to or desire for particular forms of food and drink; difficulty of swallowing and hiccough; nausea, and if there be vomiting, the character of the ejected substances; distention of the abdomen, with or without sensibility to external pressure; if sensitive, the quality of the pain produced, as of cutting, excoriation, or bruise; prolapsus of the rectum with the evacuations; tenesmus of the urinary bladder; the character of the urine voided; sleeplessness; coma; anxiety; delirium; the state of the pulse, and the like.

These are the phenomena which gather around the central, localized process of the disease, and declare its specific character; and, in so doing, point at the same time to the specific remedy which cures. Hence it is these, in our practical duties, that chiefly engage our attention. In the two aspects in which, as practical physicians, we are compelled to view diseases, first as facts in science, second as objects of our practical duties, this peripheral group of symptoms belongs by eminent importance to the latter. Though its members belong to the disease, and are as really integral parts of it as are the generic symptoms in its scientific existence and relations, still they have their highest importance in their office of guides to the selection of specific remedies. They are seldom or never all present in any one case; oftener there are but few of them, but these few are no less the guides to our choice because they are few. Whether few or many, they are our only guides to a safe and sure practice. The enlightened and conscientious physician will give no less heed to them in this case, and never, for this reason, turn from them to any, whatever, of routine resort, because Doctor this or that declares that he "cures all his cases by it." By this he simply proves that he does not know what a *cure* really is.

Etiology.—But there are other facts which pertain to dysentery in its scientific character and relations. Its etiology is the first to be considered. And of this we may remark, first, that of the specific cause of the disease, when epidemic, we know but little. Of the general circumstances and conditions which favor its development we are something better informed. The disease is found in every climate, though it is more frequent and severe in warm, and especially in tropical, lands. Its production seems to be favored by sudden and great variations of temperature. The effects of this cause are increased by such exposures as chill the body, and by which it and its clothing are made wet. And this is still further affected by continuing to wear wet clothing. The production of the disease is still further favored if to these causes are added either deficient or improper diet. When prevailing as an epidemic it attacks by preference those who are suffering from an exhausted state of the vital forces, from whatever causes. Of these, debility from the action of other diseases, excesses of any kind, protracted watching and attendance on the sick, are among the most potent predisposing causes of dysentery. While this is true as to predisposing causes, it is equally true that dysentery gives immunity to no age, sex, condition, or race, though females are said to be less frequently attacked than males, and in Europe children than adults. It may be doubted whether this last observation of foreign writers is true of the disease as it occurs in this country. My own impression is that the reverse of this is true. In my own practice I am confident that the majority of cases treated have been in childhood. For reasons above stated, the poor and destitute are especially liable to attacks, as compared with the wealthy and those whose wants are adequately provided for. As to the exact nature of the exciting cause of the disease, we have said we know but little. There are some facts, however, which may be received as settled. As early as the time of Hippocrates it was recognized that the origin of the disease was not unfrequently in some way connected with the decomposition of vegetable or animal matters—that is, with malarious effluvias. Of modern observers, *Annesley*, *Pringle*, and *Vignes* have found it to be intimately related in its origin to the miasm of intermittent fever. So many others, in different ages, have noticed the fact that now is generally received, that where these fevers are most prevalent and severe, dysenteries are most frequent and violent. While it is admitted that the two forms of disease are somehow related to the same cause, or at least are supposed to be, there has been no successful attempt to bring to the light the modifying circumstances which in any given case determine the action of this cause to result in the production of the one rather than the other.

Dysentery, like this form of fever, is in its favorite abode on southern coasts, shores of lakes, and the banks and deltas of rivers, and in countries which abound in marshes. In such localities, especially in tropical countries, the disease is found in greatest frequency and in its most violent form. The extreme in these respects is likely to be developed in the hottest months of the year and when the change of temperature from day to night is great. In all countries this last is a potent disposing cause of the disease. This is true especially in the autumn of the year, when hot days are followed by cold nights. *Annesley* found, in Bengal, the disease to be far more prevalent in those months which were hot and wet than in those which were equally hot and dry.

Errors in diet have been placed with the exciting causes of dysentery with a readiness which perhaps a more careful observation would have avoided. It is certain that this alone is not a sufficient cause. The most that can be rightfully claimed for it is, that when other influences have already prepared the organism for a ready development of the disease, this may become operative to the extent of giving the final impulse to movements which have had their initiative in other causes, and in such cases may claim to be regarded to this extent as *occasional* causes of the disease. It might be better to regard this as only a contributor of its mite to the production of the general result rather than to raise it to the importance of an independent cause, which it is not. The same is true of that other fact, *fecal stasis*, which *Cullen*, *Annesley*, *Cambay*, *Johnson*, *Martin*, and others have regarded as a cause of dysentery. *Cullen* regarded this, when the result of constriction of the intestine, as the disease itself. *Virchow* has better declared the insufficiency of this as an independent cause, and clearly states the necessity of other specific or disposing causes before this can be effectual in the production of the disease. This is, no doubt, true—so that this is rather a contributor to the efficiency of other causes than an independent agent capable of producing the general result by its own independent force.

Dysentery is met as an epidemic more frequently in warm, and especially tropical, countries than in those which have more temperate climates. But, as has been remarked, of the specific character of the epidemic causes we know nothing. We are only aware of its existence from witnessing its effects. As an epidemic, dysentery differs from cholera, yellow fever, the plague, small-pox, and some others in the comparatively limited extent of territory which it covers by a single visitation.

A potent factor in the production of dysentery is the crowding of population in great numbers into limited spaces, as in overcrowded apartments, prisons, ships, tenement houses in large cities, concentration of troops in crowded barracks, and the like, by which the atmosphere becomes loaded with poisonous exhalations from the resulting putrefying animal matters, rendering it not only unfit to support life, but a vehicle to convey and distribute the causes of disease in various forms, and these of the most destructive character. Dysentery is a frequent result of this state of things, and when so produced its severity is likely to be in the ratio of the aggravation of the cause, and of the heat and moisture of the climate and season, of the time and place.

Pathological Anatomy.—Another fact pertaining to the disease as a fact in science is the changes wrought in the tissues by its localized process in the large intestine. In other words, its pathological anatomy. These are various, the differences being determined by the severity and duration of the case. *Rokitansky* gives this description of these changes in the milder forms of the affection. The mucous membrane is thickened, and in spots reduced and injected and covered with a thin deposit, which is of a pale yellow, or grayish-red color, and of a purulent character. At the same time, if this be removed by scraping with the back of the scalpel the membrane itself is readily displaced with it, and is seen in the form of a grayish or reddish-gray bloody pulp. Here and there are found patches of greater or less extent where the mucous membrane appears reduced to a thin layer, covering the sub-mucous tissue, or this last is laid bare, the membrane having wholly disappeared. The solitary glands are swollen. The sub-mucous tissue is somewhat infiltrated, and the intestine generally distended.

In the severer forms the same author gives the following description of the changes wrought: The mucous membrane is covered with a dirty white stratum of necrosed epithelium, mixed with a purulent, thick exudation of reddish-gray color, with soft, whitish flocks, while underneath this the membrane is reddened, injected, swollen, softened, friable, and broken down in different degrees. The sub-mucous tissues, and especially the sub-mucous connecting tissue, are much infiltrated, and at the points of greatest intensity of the diseased action this last tissue appears swollen into nodules. In addition to this, the mucous membrane of the intestine here and there, and especially that of the descending colon and rectum, is covered with patches of fibrinous, membranous deposits, which very frequently, and especially on the insulated swellings just mentioned, is changed into greenish-brown, bloody, infiltrated slough, under which the sub-mucous connecting tissue appears ecchymosed. In many places the mucous membrane is at the same time eroded, with loss of the superficial stratum of its substance, the minute resulting depressions of the affected surface giving it a silverlike aspect. Besides this, on careful inspection are found excavations through the mucous membrane penetrating into the the sub-mucous tissue of the size of a poppy or millet seed. These are the result of a partial or entire destruction of the solitary glands, which have passed into suppuration.

The colon is contracted, its coats are swollen and rigid. Later it is distended and is found to contain gases, with the reddish-gray, thick, purulent matter, or a dirty brown, ichorous, stinking, flocculent fluid. The lymphatic glands of the mesocolon are swollen, congested, and softened.

In the severest forms of the disease the mucous membrane, in large connected patches, is changed into a greenish-brown, adherent, or a blackish, friable, loose slough, which is not unfrequently expelled in the form of tubular masses. The sub-mucous connecting tissue is pale, infiltrated with serum, and traversed by black branches of blood-vessels, filled with muddy, sedimentary, bloody contents. Later it is infiltrated with pus, becomes friable, easily torn, and often filled with numerous deposits of pus of greater or less magnitude, and is finally, with the mucous membrane, broken down into a black, necrosed pulp. The intestine is dilated and often collapsed, and contains a blackish-brown sediment, like coffee grounds, which smells like gangrenous matter. The veins of the mesentery not unfrequently return from the intestine a black, muddy, necrosed blood mass.

The peritoneum of the intestine in the severer and severest forms of the disease loses its shining gloss and presents a dirty, grayish color, caused by enlarged blood-vessels, and is covered with a foul, ichorous exudation, a condition which extends to the mesocolon and mesentery.

In the severest forms of the disease the intestine is not unfrequently perforated by the sloughs penetrating through the muscular and peritoneal coats, or by necrosis of the purulent infiltrated sub-mucous tissue.

Where the sloughs of the mucous membrane are followed by ulceration of the sub-mucous tissue, and this penetrates to the muscular coat, a shriveled callus results at the point of attack.

Dysentery may be either acute or chronic. When cured while in its acute form, where the mucous membrane is destroyed, it is replaced by the sub-mucous tissue or by a subjacent stratum of connecting tissue. These denuded surfaces generally extend in various directions, like threads or bands, which not unfrequently form partial or entire circles around the tube, constituting a stricture of the colon, similar to that in the œsophagus which results from the action of corrosive substances upon its mucous surface.

The surface of the cicatrix appears generally smooth, but a careful examination often shows that there are remains of the mucous membrane of the colon contained in it. In cases where the sloughing of the mucous membrane has been followed by ulceration of the sub-mucous tissue, which has penetrated to the muscular coat, shriveled, callous patches are found at the point of attack.

*Chronic dysentery* is a catarrhal inflammation of the mucous membrane of the colon, with profuse suppuration, frequently proceeding from the ulcerated follicular glands, as above mentioned, or the sub-mucous tissue becomes the seat of purulent ichorous deposits, over which the mucous membrane, in long patches, becomes necrosed. In the progress of the ulceration the sub-mucous connecting tissue is destroyed. In both varieties the ulceration extends its ramifications to the destruction of the muscular coat and the development of peritonitis and inflammation of the connective tissues of the peritoneum, especially in the neighborhood of the rectum, where abscess is sometimes the result. In the destruction of the tissues by necrosis, the process extends sometimes through the peritoneum and openings into the cavity of that sac result. The above is the pathological anatomy of dysentery as given by *Rokitansky*, than whom there is no higher authority.

Duration.—The duration of dysentery is stated by *Bamberger* to be from eight to fourteen days. Under proper homœopathic treatment it is, no doubt, much less. Indeed, it is no uncommon result of a right prescription that the disease is cut short and its existence limited to but a day or two. This can only be realized where the effects of the remedy are specifically like those of the symptoms of the case in hand. Such a remedy cannot always be found, though it should always be carefully sought for. When this search is but partially successful, cases are of longer continuance. The duration of the chronic form of the disease is very uncertain. I have had the happiness to cure a case of three years' standing, which had been contracted in India. The cure required two doses of medicine, and no more.

The same author also remarks that a perfect convalescence after acute or subacute attacks is attained only after a lapse of from one to four weeks, and is usually realized by a gradual subsidence of all the symptoms of the case; that this is seldom a sudden occurrence. This observation is also from an allopathic standpoint. Under homœopathic treatment a perfect cure is not unfrequently a short work.

He also remarks that dysentery may terminate fatally both in the acute and chronic forms. In the former, in from three to five days, from rapid exhaustion, the result of a copious exudation; from excessive evacuations and hæmorrhage, or from putrid decomposition of the exuded product and the mucous membrane, or by a perforation of the coats of the intestines. Not unfrequently in fatal cases, on inspection after death, intussusception of the ilium into the colon is found. Chronic cases are fatal chiefly through a gradual exhaustion of the vital forces by reason of the extended ulceration of the intestines and the resulting impoverishment of the blood and emaciation.

There is sometimes a troublesome sequel to dysentery in the cicatrices resulting from the healed ulcerated patches on the inner surface of the intestine. The constriction of the organ which they sometimes effect is both troublesome and dangerous. Inflammation and abscess in the cellular tissue attached to the colon and rectum is not uncommon. When met, it is always a dangerous complication. These complications belong chiefly to cases which are treated allopathically. It will be only in rare circumstances, and those of the most unfavorable kind, that they can result in cases which have been well treated homœopathically.

Diagnosis.—The distinction which it has been attempted to set up between those cases which are based on the one side on follicular inflammation and ulceration and on the other on a croupose exudation on the mucous surface of this intestine, may have a scientific interest to commend it to our attention, but practically it has not the least value: First, for the reason that the two conditions are present jointly in a great majority of the cases we are called to treat; second, because during the progress of the disease there are no such distinctive symptoms of each condition as enable us to pronounce with certainty as to the positive presence of either of these conditions to the exclusion of the other. In this connection *Bamberger* says: "The presence of a glairy mucus in lumps in the evacuations, with or without traces of blood, is an undoubted evidence of inflammation of the follicles, but it is certain that the croupose (or fibrinous) exudation may at the same time be present in every case; * * while, on the other hand, if there be clear blood in any considerable quantity, with or without a mixture of mucus, and if there appear in the stools membranous shreds or patches, or amorphous masses of exuded product, the croupose exudation prevails;" and, third, because the indices which have been made by Almighty wisdom and power, our only sure guides in the practical selection of curatives, are not found in these material conditions of the case.

There can hardly be any serious difficulty in distinguishing dysentery from other forms of disease, its phenomena are of so positive a character and their features are so distinctly marked. But it is to be remembered that all cases of bloody evacuations from the rectum are not of this family. Hemorrhages from the intestines, more or less copious, result from the tubercular, typhus, and other forms of inflammation, as well as from important congestions of other neighboring organs, or from impediments to the return of the venous circulation to the great centre from any of the various causes of this troublesome state. But these all fail of some of the essential facts requisite to bring the case within the definition of dysentery, while at the same time there are always present those facts which declare the true nature of the cause of the bloody discharge. The same remarks will apply to the hæmorrhoidal swellings and ulcerations. Cancerous and syphilitic ulcerations of the rectum may give rise to pains in the part with tenesmus, as well as to bloody discharges. But here the pain is of a fixed character, and the pus and blood are smeared over the surface of the common feculent mass, instead of constituting the principal matter voided, as in dysentery which has advanced to ulceration.

Dysentery may be complicated with many other diseases and is excluded by the presence of no other. *Rokitansky* thought it excluded by typhus, but *Prof. Oppolzer*, of Prague, in his observations upon two hundred and thirty-one cases of the disease, found it not unfrequently complicated with typhus.

Prognosis.—Under intelligent and careful homœopathic treatment and nursing, the prognosis is generally favorable. This can hardly be questioned after a practical experience in this treatment of the disease of more than forty years without the loss of a single case. In this time the number of cases treated has been great, and there is no reason for regarding them as less severe, on the whole, than the average of cases as they occur in a general practice. There are considerations, however, which are of moment always in making up our account of prognosis. Sporadic cases, in good constitutions, are the least likely to be attended with danger or to give much trouble in their cure. In different epidemics differences in the character and severity of the disease are met which may have an important bearing on the question of prognosis. Under the varied forms of treatment resorted to the mortality is found to differ greatly in different epidemics from this fact. The prognosis is affected materially by the complications with other diseases to which this is, more than some others, liable. The extent of this is determined by the nature of the complicating associate. The more grave the character of this, and the more it may have perverted the functions of the bodily organs or depressed the general standard of the vital forces, the more important its influence on the prognosis of the case. This is greatly increased where the character of the complicating element is such as has in its nature that general distinctive influence on function and tissue known as *cachexia*, such, for instance, as the tubercular or cancerous, or that which attends Bright's disease, or many of the dropsical affections, or syphilis. Patients convalescing from attacks of severe acute diseases, if attacked by dysentery, are, other things being equal, in more danger than those attacked in health. The degree of added danger is determined by the nature of the previous attack and the degree to which it has depressed the vital forces.

The surrounding circumstances of the patient may have an important influence in determining the issue of an attack. If the patient be in a crowded or badly ventilated apartment, or be subjected to the privations of poverty or to great mental anxiety, or is from any cause wanting the comforts of proper diet and nursing, the prognosis will be so much more unfavorable as any of these causes may be operative. The disease is said to be more fatal in childhood and old age than in middle life. The locality has an important influence on the prognosis; the high, dry, and freely ventilated being far more favorable than the low and marshy, and the temperate climates than the tropical. Extreme heat in either is unfavorable.

for the solution of this question; to "Gotham," of late so prolific in medical discoveries.

What are, then, the future possibilities after this certainly secured success? The newly discovered laws and the practice based on them will prevail, Hahnemann will be forgotten, a new school will rise, and people will recover so rapidly that the sick will require but very little medical advice. Homœopathy and allopathy will be forsaken alike, and an enterprising "Gothamite" will take out a patent for speedily potentizing any and every morbific or other unproved substance; every one of the few remaining doctors will run his own "potentizer" day and night; all pharmacies will be closed, and the discoverer will be immortalized! Such are the possibilities of the future; not only the possibilities but the certainties in the near future, *provided* the practical results of applying the new method warrant it.

For ourselves, we can only say to the discoverer that we will be happy to make the experiment just as soon as Homœopathy, such as its founder taught us, ceases to show us the best and only means to cure the curable sick and relieve the incurables better than any other system in vogue. And if that time has come, and if by the subsequent practical experiments we become convinced of the great superiority of successes under these new discoveries, our first duty will be to denounce Homœopathy, cease our connection with all and every so-called Homœopathic Society or Association, and become a member of a society of men who have learned to cure the sick so very rapidly with so little waste of time both to the doctor and the sick. The ONE case has given us food for reflection; that ONE case deserves a prominent place in every homœopathic journal in particular and in *all* medical journals in general; and that ONE case will make *all* medical men "*reflect.*" This ONE case will stand there before the medical world unsurpassed in the novelty of therapeutics until even this *celebrated* case has been surpassed by still more rapid cures with much smaller quantities of more intensely potentized, unproved drugs. Reflect, that every great thing once accomplished can and must be accomplished again, and the reflecting reader will ultimately, if not at once, see the deep philosophy, the profound reasoning, the extraordinary skill brought to bear on this *celebrated case.* By all means let us progress and investigate.

*Libertas est protestas faciendi id quod jure licet.—Cicero.*

### OVERTAXED BRAINS.*

### A PHILOSOPHICAL AS WELL AS MORAL LESSON FROM A SCIENTIFIC STANDPOINT.

R. R. Gregg, M. D.

The gentleman referred to in the following article was the noted pianist and composer of our city, Mr. Alfred H. Pease. He had always led a very exemplary life and worked excessively hard in his profession—so much so that he commonly had a half-dazed expression of countenance. He went to Chicago in May last and played a very successful engagement for a week, then went or wandered off to St. Louis, where his friends lost all trace of him for several weeks, until his dead body was found under the following circumstances: A man was seen to fall in the streets of that city one day and died soon after. An inquest was held, but no knowledge obtained as to who he was until a reporter accidentally recognized his body at the Morgue as that of Mr. Pease, from the descriptions he had read of him. Then it was learned he had been on a debauch there for weeks, and from all accounts the first in his life. He had evidently lost all moral control of himself through his excessive intellectual efforts for years and through the physiological process pointed out in the course of this article. What a pity, therefore, he could not have been found and properly cared for until restored to his former self again!

In an editorial upon the fate of Mr. Pease in Sunday's *Courier* of July 16th we read: "He was, indeed, almost the last man under the sunny exterior of whose nature one would have suspected a lurking mystery of evil. Let the sod cover his faults, and his fate and his memory survive only in his music." The words in this quotation, "lurking mystery of evil" and "let the sod cover his faults," I take as the text of a discourse which I have long felt should be written by some one who could give us something true to nature, or, at least, a modicum of truth upon the subject of overtaxed brains, and which, from not a little individual experience and no one appearing to undertake the task, I feel it a duty to essay now myself, even though it may be done in a very imperfect and unsatisfactory manner.

I was not acquainted with Mr. Pease, excepting to know him by sight, and knew nothing even by hearsay of his daily habits or of occasional harmful indulgencies, if, indeed, he were guilty of such; therefore, I do not feel called upon to defend his memory either on

* Published in Buffalo *Courier*, July 30th, 1882. Revised for re-publication.

the score of acquaintance or from knowledge of facts creditable to his daily life. But I take up the subject on a much broader ground and take him as a type of a large and rapidly increasing class of mentally overworked men who, in their acts, if not otherwise, are daily calling aloud for help, and should receive it. "They know not what they do," and should be instructed in the right. In other words, they should be given a clear and simple philosophy on the subject of mental overwork by which they can guide themselves before it is too late, or by which others can guide them when they have gone too far.

From the little knowledge I did have of Mr. Pease, and simply in his capacity as a prominent musician and composer, I take it that his most harmful indulgencies (harmful to himself, I mean) were in the excessive study to perfect himself and excel in his chosen profession. Judging him by sight and by his accomplishments in his art, his mind was wholly absorbed in that direction to the exclusion of almost everything else.

This exclusive devotion to one thing or to one line of thought year after year is always very wearing, as all know; but it is also the rock on which many a devotee is *morally* wrecked, which few or none seem to know.

To illustrate: Whether phrenology be true or not, there are three great divisions of the human mind which all recognize, namely, intellectual, moral, and immoral or animal. And it may be safely assumed that each of these manifestations of mind has its special seat of activity in or depends upon some special division of the brain. Indeed, it has come to be generally regarded that the anterior or frontal brain is the seat of intellectual action, the superior or top brain the seat of the moral sentiments, and the posterior and base of the brain the home of animal forces.

Again, it is certain that the blood carries to and furnishes the brain with the nutriment upon which the various operations of the mind depend. If there were no activity of nutrition in the cells of the brain there could be no activity of thought, whether good or bad. And if no blood were carried to the brain there could be no activity in the nutrition of its cells, which is a negation of thought.

This, then, leads us to the inquiry: What part of the blood nourishes the brain? The answer is, a portion of the fatty matters and a portion of the salts. How much of these are there in the blood? Why, there are not to exceed *eight* parts of fatty matters and salts combined in *one thousand parts* of blood (less than one part of both in one hundred of blood), and yet even this little cannot all be used in the nutrition of the brain, as a portion of both must be used for other purposes. The salts must be used in part for the nutrition of the bones and also somewhat for the muscles, etc., while the fatty matters must be partly used in deposits to facilitate the movements of parts upon each other and as cushions to other parts, also as a covering for protection to organs like the bowels against cold, etc., etc. (And here let me say, in passing, that the reason why very active intellectual people, especially those of quick, exhausting, nervous activity, are so commonly spare, or at least not corpulent, is because they use up so much of the fatty matters of the blood in the brain that not enough of them are left for deposits to make them plump, or for protection against cold, hence such are generally so easily chilled.)

Now for the application of the above facts to our moral natures: Supposing a man like Mr. Pease, any man, in fact, to apply himself so excessively to one or a few lines of intellectual thought that he uses up all the brain-nourishing material of the blood in the intellectual part of the brain and leaves none of it, or a very insufficient supply, to sustain the moral part of it, what must be the consequences? Why, in that case, of course, the moral organs are starved, consequently cannot put forth any vigor of moral sentiment, which leaves the victim for the time being more or less completely without a moral guide, until nature can have time and opportunity to establish a reaction that will set things going on again in their normal course; that is, until the moral organs are allowed to receive their full proportion of food again and be set right thereby. But here is another highly important point to be considered. Nature always takes care of herself first; that is, her greatest concern and effort is to save the life of the individual first, leaving wrongs against her better work to be rectified afterward. And as life depends upon the activity of the animal forces, which have also in these cases been robbed of their support along with the moral organs, and she being unable, as things are constituted, to preserve life except through the animal parts of the brain, she arouses these to activity first; that is, she feeds them before she does the moral organs, and it is during this interim, be it long or short, that the one who has thus so excessively overtaxed his brain intellectually is necessarily deprived of the requisite moral support within himself and the animal has sway. And then is when he should have the proper moral support from without, and from those who know, or should know, *what they are about and what to do.*

Here, then, is the "lurking mystery of evil" that may be developed in almost any of us in that way; the "fault" that the sod, or the mantle of charity, should cover so deeply in such cases that not the slightest allusion should ever be made to it afterward in censure, in ridicule, or in jest, and not even in earnestness, unless it were to raise the note of warning in time to prevent the transgressor, if he still lived, from a repetition of the causes of his misfortunes in subsequent long-continued, exhausting mental work.

The "fault," therefore, if fault there be, is in the original transgression (or in violation of the harmony which nature has set up in all moral minds), by such excessive application to intellectual labor that the moral part of the brain is robbed of the sustenance required to enable it to act with vigor and hold control; hence the temporary lapse to purely animal indulgences, as shown, until the wrong is righted. But the victim knows nothing of a moral wrong being involved in that original step. Indeed, who does? The ignorance that prevails upon the subject in this light is something worse than "Egyptian darkness." The books upon mental and moral philosophy, upon psychology and insanity, which have fallen under the writer's observation are as innocent of any such teaching upon their respective themes as unborn babes are of such conceptions. Authors and readers alike are equally ignorant; yet who shall say that the simple facts here presented should not be made at once the cornerstone of a new philosophy upon the subject, by which all may be guided and through which all may be saved?

Who now thinks that any moral wrong, or even harm, can come, excepting to the individual's physical health, from excessive mental application in any of the great elevating walks of life? All think that the more excessive the application the more commendable the effort, so long as the physical health holds out, little knowing that the worst results, or even actual crimes, may come of it; if, indeed, we may call the resulting acts crimes.

But nature is ever kind, or ever tries to be, in avoiding or trying to avoid by various devices such immoral extremes being reached, except it be after years of great transgressions, and even then, if left to herself or not drugged, she often succeeds in her purposes against the wrong. For instance, she sets up all kinds of counter-actions to tide over the emergency. She permits of the exhaustion of the intellectual forces and thus stops the transgressor, by rendering him unable to think or study, until time has been given for a recuperation to come that will restore the exhausted brain to its normal condition before the moral forces have been reached to be seriously harmed. She forces sleepiness and gets recuperation through sleep in the early stages of such cases. Or, when the violator has pushed his violations too far and overcome that obstacle to his work, then she sets up sleeplessness to further exhaust and stop him in that way. She disgusts him with his subject and thus gets time for repairs. She often excites severe headache to stop the excessive work. She establishes great irritability of mind to thereby divert him from his purposes, and here the animal steps in to try and right things. She sometimes arouses great fear through unnatural and strange sensations and stops him in that way. She quite commonly sets up physical diseases as a consequence of the mental efforts depriving the blood too rapidly of its fatty matters and salts (and thereby destroying its normal constitution, that is, the proper proportion among its constituents), until physical disease or physical pain is produced to divert his attention. This applies very commonly to the earlier violations in this direction, as in school children who are urged on to excessive study to their great injury (which it is hoped both teachers and parents will deeply consider), although it is a frequent result to older transgressors as well. And last, but not least, she very commonly and more or less fully destroys hope for the time being, through that part of the brain which is the seat of our hopeful natures being starved so that it cannot act, and thus produces the "blues," or melancholy, to thereby stop the too close and excessive mental application.

But for these various devices being set up for the purpose stated, the moral and physical wrecks of men from mental overwork would be much more numerous than they are now, although now far too numerous and rapidly increasing. Indeed, but for such guards over us, many men would be destroyed where one is now, and few or none who become so wholly absorbed in any intellectual pursuit would be safe from moral degradation, from suicide, from insanity, or from premature death through disease. Having had not a little personal experience, as well as some professional observation, in many of these efforts of nature to avert worse results, the writer feels that he knows something of what he speaks and has not overdrawn the picture.

Such, then, is nature's intelligent and practical way of dealing with this subject. Let us now contrast with it man's ignorant and stupid way of trying to cheat nature out of her reasonable and just demands and stop her restorative efforts. Among the most common devices of man to right this wrong against nature is the resort to alcoholic stimulation. But, alas for the victims who get started in this direction! Three-fourths or more of them are sure of moral wreck. In addition to the mastery that alcohol is liable to get through the appetite over every one who indulges therein, it stimulates the intellectual faculties to greater work for the time being than they could otherwise perform, thereby using up more of the fatty matters and salts of the blood in the intellectual organs and starving the moral organs more than nature would otherwise allow. Matters then go on from bad to worse, more work and more drink "to keep up on," until the moral sentiments are lost through simple want of food to sustain them, and the subject has lost all self-control and becomes a sot in spite of anything he can do to avert it. And here nature again tries to save herself and the victim through the inactivity and stupidity that excessive drinking brings to cut off all mental action, but the appetite has become so strong while under the loss of moral control that it is then often impossible, or nearly so, to subdue it.

The writer has never had this part of the experience, as he knew beforehand what must come of it and shunned it; but he has seen times that he would almost have given worlds, if possessed of them, for relief.

As bad or worse off still is the victim that falls into the hands of a physician who knows little or nothing of these things, and does not appreciate the great efforts nature is making through sleeplessness, depression, melancholy, and other ways to save his patient, but drugs him with morphine, chloral, bromides, stimulants, etc., as the case may be, until all her efforts are paralyzed and she loses the mastery. The writer was once told by a medical superintendent of an insane asylum that during the years he had charge thereof in not one instance where the patient had been freely drugged with the agents named before his admission was he cured, whereas those not so drugged were quite a large proportion of them cured. Softening of the brain, paralysis, apoplexy, or other physical disease and wreck are often also as legitimate results of such drugging as are imbecility, suicide, or incurable insanity, all of which are commonly produced by it.

When will physicians see and learn that the *natural* road back to a healthy and vigorous moral activity of those who are naturally moral, but have lost control in the ways named, is through depression, melancholy, and other more or less similar means? "Suffer and be strong," through nature's vigorous reactions, is certainly the motto here, unless the suffering is carried too far and unless nature is stopped in her beneficent work by drugs. And here let me tell the sufferer that his clearest and best thoughts will come after such suffering and reaction, if he has not given cause for the former to be too great, and he be not drugged. Drugging, however, will destroy subsequent clear thoughts for days, if not weeks, and make the ultimate suffering much greater than it otherwise would or could be. If the suffering becomes too great there are other ways of relieving it than by drugging with the above-named agents.

If any should doubt that through irritability, extreme nervous sensitiveness, melancholy, etc., lies the royal road back to normal conditions in many cases, let me cite them the almost universal fretfulness, crossness, and irritability of children when over-tired from play, or when recovering from sickness, as the proof. The universality of this process of nature in restoring children to normal conditions proves what it means, and shows us of what great value a proper application of the fact to the guidance of the mentally overworked might be. The child could not be restored to health and vigorous activity when seriously overcome by excessive efforts, or disease, without going through the reactive efforts named; the mentally overworked cannot get back to their proper moral basis without melancholy and other efforts at reaction; then how wrong to stimulate, drug, or otherwise destroy this, the only process of restoration!

Still further, if any doubt that sleeplessness is one means of restoration to a greatly overworked brain, let me tell them of a little individual experience. Often has the writer lain awake at night, hours upon hours together, with thoughts running at racehorse speed in consequence of the blood having been drawn in such great excess to the thinking organs to sustain a prolonged intellectual effort, until finally exhaustion would follow, or the excitement exhaust itself; then an aching of the limbs would arise to tell of the coming reaction; then profound sleep, a few hours of which would restore the exhausted mental energies and vigor of thought the same as before. And there was where a halt for a day or two ought to have been called, each time, to have saved much more severe subsequent suffering, but was not. Supposing, on the contrary, an anodyne had been taken to force sleep and thus stopped the natural process of reaction, this would not have come in days, if in weeks, and the sufferings to get out from under the effects of the anodyne would have been much greater than the lying awake; while under a frequent repetition of this violation of nature the writer would not have been here to have told his story, or would have been rendered incapable thereby of telling it.

In conclusion, the facts, or claimed facts, on moral issues, given in this discourse, must not be held to excuse the naturally depraved in their wrong-doing, or to tolerate their continuance in depravity; but to those who have exhausted themselves in *well-doing* until immoral acts (but generally to themselves) have resulted, such facts afford a rational basis for excuse until the transgressor can be restored to his proper and natural moral basis. And it is hoped that this discussion of the subject may enlighten all more or less in the matter and help not a few who are now suffering, or those who may suffer in the future, to a clearer understanding of their cases, and thereby aid them in getting back to their former selves again.

---

### NOTES ON "AFTER-PAINS."

ACTEA RAC.—Crampy pains in groins; pains cause flushing of face; predisposition to neuralgia, which seems to be reflex from uterine disorders.

CAULOPH.—Pains spasmodic, felt in uterus, bladder, and reflex pains in chest and back; patient nervous, weak, sleepless.

CUPRUM.—In tedious after-pains in those who have had many children.

USTIL AGO.—Lochia too profuse, partly fluid and partly clotted; prolonged bearing-down pains; uterus feels as if drawn into a knot.

XANTHOX.—Pains excruciatingly severe; pains extend down along genito-crural nerves.—Dr. Farrington in *Am. Jour. of Obstetrics, etc.*

### REST AS A CAUSE OF AGGRAVATION.

Dr. C. Von Bœnninghausen.

All that needs to be said concerning the influence of *rest* upon the aggravation of symptoms may be done with considerable brevity, for it is essentially the converse of what has been said regarding *motion*.

One variety of rest, however, demands a brief consideration, and all the more because, in the first place, it affords for many internal and external diseases a truly indispensable characteristic, and, in the second place, because it must astonish every experienced homœopathist to see, as in the more recent descriptions of clinical cases, that many practitioners appear to leave it altogether out of account. I refer to *rest* in a *recumbent position.*

I pass by *simple* lying, which is merely rest in contradistinction to motion, and also *lying in bed*, and propose to consider the *different recumbent positions*, which are points of greatest interest in this connection.

First under this comes aggravation from *lying outstretched* in contradistinction to *lying crooked* or in a *curved position.* For aggravation in the former posture, Cham., Colch., Platina, Puls., Rheum, Rhus, and Staph. are most likely to be appropriate. For aggravation in the latter, most frequently, Hyos., Lyc., Spong., Teucr., and Valer.

Aggravation from *lying with the head low* indicates other remedies again, among which are Ant. t., Arg., Ars., Caps., China, Colch., Hepar, Lach., Nitrum, Puls., and Spig. If in addition the horizontal position is most tolerable to the patient, then Apis, Arn., Bell., and Spongia may be added to the above.

But still more important than these are the recumbent positions upon the back and upon the *sides.*

If *lying upon the back* aggravates, this indicates especially Amm. m., Ars., Caust., Cham., China, Coloc., Cupr., Cycl., Iod., Nitr., Nux v., Phos., Plumb., Rhus, Sep., Sil., or Spig. When, on the contrary, this position affords relief, the most suitable remedy will generally be found among Acon., Anac., Bry., Calc., Carbo an., Kali c., Lyc., Merc., Puls., Seneg., Stann., Thuja.

Aggravation from *lying on the side* requires in *general* Acon., Anac., Bry., Calc., Carbo an., Kali c., Lyc., Phos., Puls., Stann., Sulph., and Thuja. But under this head there are two varieties which are of very great importance, viz.:

sensation as if broken; great heat and thirst, with redness of the face. The importance of this drug in the treatment of dysentery is hardly second to that of any other. That is to say, the proportion of cases in practice which call for this remedy is as great, to say the least, as that which shows relationship to any one other drug. The resemblance of the specific symptoms by which this and one or two other important remedies are related to the treatment of this disease is so great that to the beginner there is often no little difficulty in deciding as to which the preference is to be given in a particular case. Take, for example, *Caps.*, *Merc. sol.* and *Nux vom.* and we have a group of remedies equal to the cure of a large majority of cases as they occur in this latitude. But it is by no means a matter of indifference which of the group we shall give to any one case, or whether, indeed, we shall give either of them. The difficulty of selection between these three is chiefly in the great resemblance of their symptoms. This is so great that many have been left to the only resort they knew, that of giving them in succession, if the right have been missed. This ought not to be and need not. Care on the part of the practitioner and memory of the distinctions we are about to give will make a mistake impossible. This is here the more to be insisted on, as the use of the wrong remedy not only protracts the torture of the patient, but renders his cure more difficult, if not uncertain, by the use of that which is right subsequently, a danger we cannot too carefully avoid.

These drugs are alike in the character of their evacuations, the quantity of each being small with each discharge. The discharges of each are preceded by similar severe pains, which are continued through the period of the evacuation. In all they are attended by severe tenesmus. In all they recur at short intervals, with pains *extending to the back.* The above are the similarities. The following are the differences: With *Nux vom.* the pains, especially those in the back and the tenesmus, *cease with the evacuation.* Those of *Caps.* and *Merc.* are continued *after.* The pains of *Nux v.* in the *back* are pressing, as if broken, and like a bruise. Those of *Capsicum* are drawing. Those of *Merc.* like a bruise. With a recollection of these facts and a careful attention to the symptoms given of each of these remedies, there need be no confusion in prescribing them because of their resemblances.

*Plumbum* has great violence of tenesmus; frequent and almost fruitless efforts to stool; cutting pains, with violent outcries; retraction of the abdomen; constriction and retraction of the anus. *Prolapus ani.*

*Pulsatilla* belongs rather to dysenteric diarrhœa than to true dysentery, but may be appropriate in cases with slimy evacuations, slight tenesmus, and nocturnal aggravations.

*Rhus tox.*—This remedy is rarely called for in the early stage of the disease, but is often valuable later in the attack, especially when there are nocturnal exacerbations, and also in the diarrhœas which sometimes follow dysentery. The case is marked for *Rhus* if there be constant tenesmus and urging to stool, with nausea and the passing of but little bloody water. It is appropriate also in the late stage of the attack, with nocturnal aggravations.

*Sulphur* has spasmodic constricting pains, extending to the chest, groins, and genitals; cutting pains while urging at stool; from pressure on the abdomen or bending the body backward; *prolapsus ani* at stool; cutting pains in the abdomen, lower part of the loins, and upper part of the sacrum after midnight; pains relieved by the application of dry heat to the abdomen; the blood in the stool is in streaks. *Sulph.* is especially appropriate in cases attended with difficult breathing at the outset and also in those of hæmorrhoidal subjects. It is seldom in place at the commencement of an attack, but in the later stages is often of great value, and even at times indispensable, especially in cases threatening ulceration of the mucous surface of the intestine. When this great evil has actually occurred *Sulph.* is still one of our chief reliances for a cure, and in this state is related to *Arn.*, *Ars.*, *Lach.* and *Merc.* In selecting either of these remedies in a given case careful attention is to be given to the specific symptoms of the case and the drug before the decision is made, and of these the general or constitutional symptoms are to be regarded as of the first importance. There are, to be sure, differences in character of the discharges of these drugs which are to be noted, but they are not more important than those general and too often overlooked symptoms which in many cases are the only sure guides to the true remedy.

A review of the symptoms of the different remedies we have given above, with a recollection of what we have said of generic and specific symptoms of the disease and the drug, will make easy a clear understanding of the true nature of the specific treatment of this and other diseases. It will be seen in these groups of the symptoms of the different drugs that there is little of resemblance between those of one drug to those of either of the others. There are almost no repetitions of the symptoms of one drug in those of any of the others. This makes certainty of knowledge of the true remedy, under the guidance of the law of similars, a comparatively easy matter. That which is like in the disease to either of these groups is not like to either of the others. Look at the symptoms of the three first remedies in the list—*Aloes*, *Arn.* and *Ars.* There is no resemblance. Take the generic symptoms of this disease as they stand represented in the same drugs, and the resemblance is so great as to make uncertainty inevitable. It is still worse if we take that other most important group in the treatment of dysentery, *Caps.*, *Merc.* and *Nux v.* It is impossible to distinguish between these symptoms as they are found in the pathogenesis of these drugs. But with the peripheral or specific symptoms there is no difficulty at all, and it is chiefly under the guidance of these, according to the law of cure, that we secure the greatest safety and success.

